# Quantification and characterization of *Salmonella* spp. isolates in sewage sludge with potential usage in agriculture

**DOI:** 10.1186/s12866-014-0263-x

**Published:** 2014-10-22

**Authors:** Flávio Krzyzanowski, Lincohn Zappelini, Solange Martone-Rocha, Milena Dropa, Maria Helena Matté, Flávia Nacache, Maria Tereza Pepe Razzolini

**Affiliations:** Instituto Federal de Educação, Ciência e Tecnologia de São Paulo - Brasil, Rua Pedro Vicente 625, 01109-010 São Paulo, Brazil; School of Public Health of University of São Paulo-Brazil, Av. Dr Arnaldo 715, 1°andar, 01246-904 São Paulo, Brasil

**Keywords:** *Salmonella* spp, Sewage sludge, Antimicrobial resistance, Plasmid presence, Virulence genes presence

## Abstract

**Background:**

This study aims to scrutinize *Salmonella* spp. and its serotypes in sewage sludge samples from wastewater treatment plants, and assesses the presence of virulence genes and antibiotics resistant to the profile. Samples (n = 54) were collected and analyzed in accordance with the EPA Method 1682/2006. For positive serological reaction, 40 strains were selected for PCR analyses and detection of s*pvC*, *invA* and s*seL* virulence genes, plasmid presence and resistance to antibiotics.

**Results:**

*Salmonella* spp. was detected in 38.9% of the samples collected (<0.006473 to 12.19 MPN/gTS). The most prevalent serotype was *Salmonella* Infantis. All *Salmonella* spp. (n = 35) presented at least one of the three virulence genes mentioned above and 40% harboured plasmids. *Salmonella* Typhimurium strains were isolated harbouring at least one of the following virulence genes: s*pvC*, *invA* or s*seL*. Four *Salmonella* spp. isolates were resistant to tetracycline; three were resistant to trimethoprim-sulfamethoxazole, and one isolate was resistant to ciprofloxacin. Two *Salmonella* spp. strains presented multi resistance to antimicrobial agents.

**Conclusions:**

The results obtained demonstrated that *Salmonella* spp. have been found in sewage sludge, thus it is essential to set measures to mitigate human health risks when it is intended to be applied on agricultural soils.

## Background

The employment of sewage sludge on agricultural fields has been recognized worldwide as a promising way to manage this kind of residue, as it can minimize environmental pollution, in addition to giving it useful destination, what is done at the Wastewater Treatment Plants (WWTP). None the less, careful consideration regarding its quality and impacts on human health ought to be given, since the presence of pathogens in sewage sludge, including *Salmonella* spp., have been documented in manifold studies [1-4].

In order to protect both human and environmental health, Brazilian legislation issued by Conselho Nacional do Meio Ambiente (Environmental National Council), which is a branch of the Federal Environmental Council, CONAMA N^o^ 375/2006 – has classified sewage sludge in two distinct classes: A (unrestricted application) and B (restricted application). For class A, the maximum values for thermotolerant coliforms is ≤10^3^ MPN/gTotal Solids (TS), the total absence of *Salmonella* spp. in 10 g of total solids, followed by <0.25 helminth viable ova/gTS and ≤0.25 enterovirus/g TS, while for class B the standards are ≤10^6^ MPN/gTS for thermotolerant coliforms and <10 helminth viable ova/gTS [5]. Some countries or some of their respective regions have been establishing microbiological and parasitological standards for biosolids, such as the USA legislation [6], the United Kingdom [7], Australia (South Australia, West Australia and EPA Victoria), Finland and New Zealand [8].

The employment of sewage sludge with no sanitary criteria or barriers, particularly in agricultural fields, intensifies the potential to disseminate such pathogen.

Of late, a worldwide increase in the number of outbreaks involving *Salmonella* spp. related to the fresh-cut produce industry and the consumption of fresh-cut vegetables has been observed. Such outbreaks are caused by a few *Salmonella* serotypes which have the capability to resist to environmental stresses, remaining viable for extended periods on field crops [9-11]. It has been observed that *Salmonella* can regrow in the soil and remain viable for more than two years after soil inoculation [12].

In a global survey conducted from 1990 to 1995, *Salmonella* Enteritidis, and *Salmonella* Typhimurium were considered to be the most isolated non-typhoid *Salmonella* in fifty-nine countries [13]. None of the less, in the city of São Paulo (Brazil), where surveillance studies indicated an increase in the prevalence of *Salmonella* Enteritidis both in clinical and environmental samples, whereas studies conducted in Agona, Ohio and Albany demonstrated the most isolated serotypes from sewage samples [14,15].

The presence of virulence genes such as *invA* and *sseL*, which are located, respectively, on the pathogenicity island I (SPI-1) and II (SPI-2), has been used as evidence of pathogenicity [16]. The virulence genes which compose SPI Pathogenicity Island are responsible for the entry of *Salmonella* spp. into host epithelial cells. The pathogenic genes presented in SPI-2 are related to modifications in host cell functions, being thus essential for survival and replication of *Salmonella* Enterica within host macrophages [17]. The *spvC* virulence gene, present in plasmids and/or chromosomes, strengthens the systemic proliferation of the pathogen and, furthermore, contributes to its replication in extra-intestinal sites [18]. The presence of these three virulence genes predicts overall pathogenicity, invasiveness and replication capabilities of *Salmonella* species.

Antimicrobial resistance patterns for typhoidal and non-typhoidal *Salmonella* are constantly changing, and the treatment of infections caused by multidrug resistant strains (MDR) poses a serious public health concern. A study conducted in Brazil with *Salmonella* spp. strains isolated from clinical specimens which were collected in each of the five Brazilian regions, detected that this microorganism presented low resistance to all antibiotics tested, except for cloranfenicol [19]. Later on, a study conducted by Medeiros *et al*. [20] with the participation of several Brazilian states, pointed out to the fact that all the 250 *Salmonella* strains which were previously isolated from chicken carcasses were resistant to at least one antibiotic, and that 53.2% of the samples assessed were MDR.

In the light of the fact that the use of sewage sludge on agricultural fields can be considered a way to spread such pathogen, and therefore risk the health of the population exposed, the aim of this study was to survey *Salmonella* spp. and its serotypes in sewage sludge samples from five WWTPs located in São Paulo metropolitan region (MRSP). In addition to so, we intended to search for the presence of virulence genes, plasmids, and antimicrobial resistance/resistant profiles in the isolated strains.

## Results

In total, 54 samples were collected from the five WWTP sites throughout the 12 months of 2011, and analysed in order to observe *Salmonella* spp. presence. Table 1 shows *Salmonella* concentrations in sludge samples from each WWTP studied from January to December 2011. This bacterium was detected in 38.9% (21/54) of all samples analysed. Bacteria concentration above <0.006473 MPN/g TS (Detection Limit - DL) was detected in 38.9% (21/54) of all samples analysed. The highest concentration of *Salmonella* spp. was found in WWTP 2, where counts ranged from DL (<0.006473 MPN/g TS) to 12.19 MPN/g. The lowest *Salmonella* spp. concentration was found in WWTP 1 (0.75 MPN/g) and WWTP 5 (0.43 MPN/g), and no *Salmonella* spp. was isolated in WWTP 3.

**Table 1:** **Concentration of** ***Salmonella*** **spp. (MPN/g TS) in sewage sludge samples collected from the five WWTPs in 2011**

**Date**	**WWTP 1**	**WWTP 2**	**WWTP 3**	**WWTP 4**	**WWTP 5**
Jan	<0.006473	<0.006473	<0.006473	0.43	<0.006473
Feb	<0.006473	1.74	<0.006473	0.49	0.43
Mar	<0.006473	3.72	NP	<0.006473	<0.006473
Apr	<0.006473	6.03	<0.006473	0.21	<0.006473
May	<0.006473	4.81	<0.006473	<0.006473	<0.006473
Jun	<0.006473	5.63	<0.006473	NP	<0.006473
Jul	<0.006473	2.28	NP	1.32	<0.006473
Aug	<0.006473	12.20	NP	<0.006473	<0.006473
Sep	<0.006473	3.29	NP	0.69	<0.006473
Oct	<0.006473	1.66	<0.006473	0.76	<0.006473
Nov	0.75	5.74	NP	0.85	<0.006473
Dec	<0.006473	4.45	<0.006473	1.86	<0.006473

NP = Not Performed.

Table 2 summarizes the results obtained via serology identification. Such results indicate the presence of virulence genes, plasmids, and antibiotic resistance in *Salmonella* strains which were previously isolated from sewage sludge. Out of the 307 isolations, 40 were selected and subjected to serological typing. Five strains could not be serotyped. The most prevalent serotype among the strains which were submitted to serotype procedure was *Salmonella* Infantis, where 6 out of the 35 strains analysed could be classified as such, followed by *S.* Agona with 14.3% (5/35), and 8.6% (3/35) in regard to *S.* Anatum, *S.* Corvallis, *S.* Mbandaka, and *S.* Typhimurium; 5.7% (2/35) in regard to *S.* Müchen, *S.* Salamae, and 2.8% (1/35) in regard to *S.* Give, *S.* Javiana, *S.* Minnesota, *S.* Molade, *S.* Newport, *S.* Ohio, *S.* Oranienburg, and *S.* Ouakam.

**Table 2:** **Serological identification, presence of virulence genes, plasmids, and resistance to antibiotics regarding** ***Salmonella*** **strains**

**Sample source**	**Serovars**	**Presence of virulence genes**			**Plasmid**	**Antibiotic resistence**
		***invA***	***sseL***	***spvC***		
WWTP 2	Infantis	+	+	-	+	S
WWTP 2	Infantis	+	+	-	-	S
WWTP 2	Infantis	+	+	-	-	S
WWTP 2	Infantis	+	+	-	-	S
WWTP 4	Infantis	+	+	-	-	S
WWTP 4	Infantis	+	+	-	-	S
WWTP 2	Agona	+	+	-	-	S
WWTP 2	Agona	+	+	-	-	AMP,CIP
WWTP 2	Agona	+	+	-	-	S
WWTP 2	Agona	+	+	-	-	S
WWTP 4	Agona	+	+	-	-	S
WWTP 2	Anatum	+	+	-	+	TET,SUT
WWTP 2	Anatum	+	+	-	+	TET,SUT
WWTP 2	Anatum	+	+	-	-	S
WWTP 4	Corvalis	+	+	-	+	S
WWTP 4	Corvalis	+	+	-	+	S
WWTP 4	Corvalis	+	+	-	+	S
WWTP 2	Mbandaka	+	+	-	-	S
WWTP 4	Mbandaka	+	+	-	-	S
WWTP 4	Mbandaka	+	+	-	-	S
WWTP 4	Typhimurium	+	+	-	+	TET, AMP, SUT
WWTP 5	Typhimurium	+	+	+	+	S
WWTP 5	Typhimurium	+	+	+	+	S
WWTP 1	München	+	+	-	+	CAZ, TET, ATM, CTT, COM
WWTP 2	München	-	+	-	+	S
WWTP 4	Salamae	+	+	-	-	S
WWTP 4	Salamae	+	+	-	-	S
WWTP 4	Give	+	+	-	-	S
WWTP 4	Javiana	+	+	-	-	S
WWTP 2	Minnesota	+	+	-	-	S
WWTP 2	Molade	-	+	-	+	S
WWTP 4	Newport	+	+	-	-	S
WWTP 2	Ohio	+	+	-	+	S
WWTP 2	Oranienburg	+	+	-	-	S
WWTP 2	Ouakan	+	+	-	+	S

(+) presence, (-) absence; S-susceptible; AMP: ampicillin; CTT: cefotetan; TET: tetracycline; CIP: ciprofloxacin; SUT: sulfametoxazol; CAZ: ceftazidime; ATM: aztreonam; COM: cefepime.

The results referring to the detection of virulence genes revealed that all *Salmonella* spp. isolated strains previously analysed presented at least one of the *spvC*, *invA,* and *sseL* genes. Two strains (*Salmonella* Molade and *Salmonella* Muechen) presented negative results for *invA* gene, and two strains of *Salmonella* Typhimurium present at the *spvC* gene. From the 35 isolates subjected to plasmid extraction, 14 (40%) presented at least one plasmid.

Antimicrobial susceptibility tests revealed that 14.2% (3/35) of the strains were resistant to two antimicrobial agents, and just two strains 2/35 (5.7%) were MDR. All isolates were susceptible to cefotetan, imipenem, meropenem, and ertapenem (Table 2).

**Figure 1:**
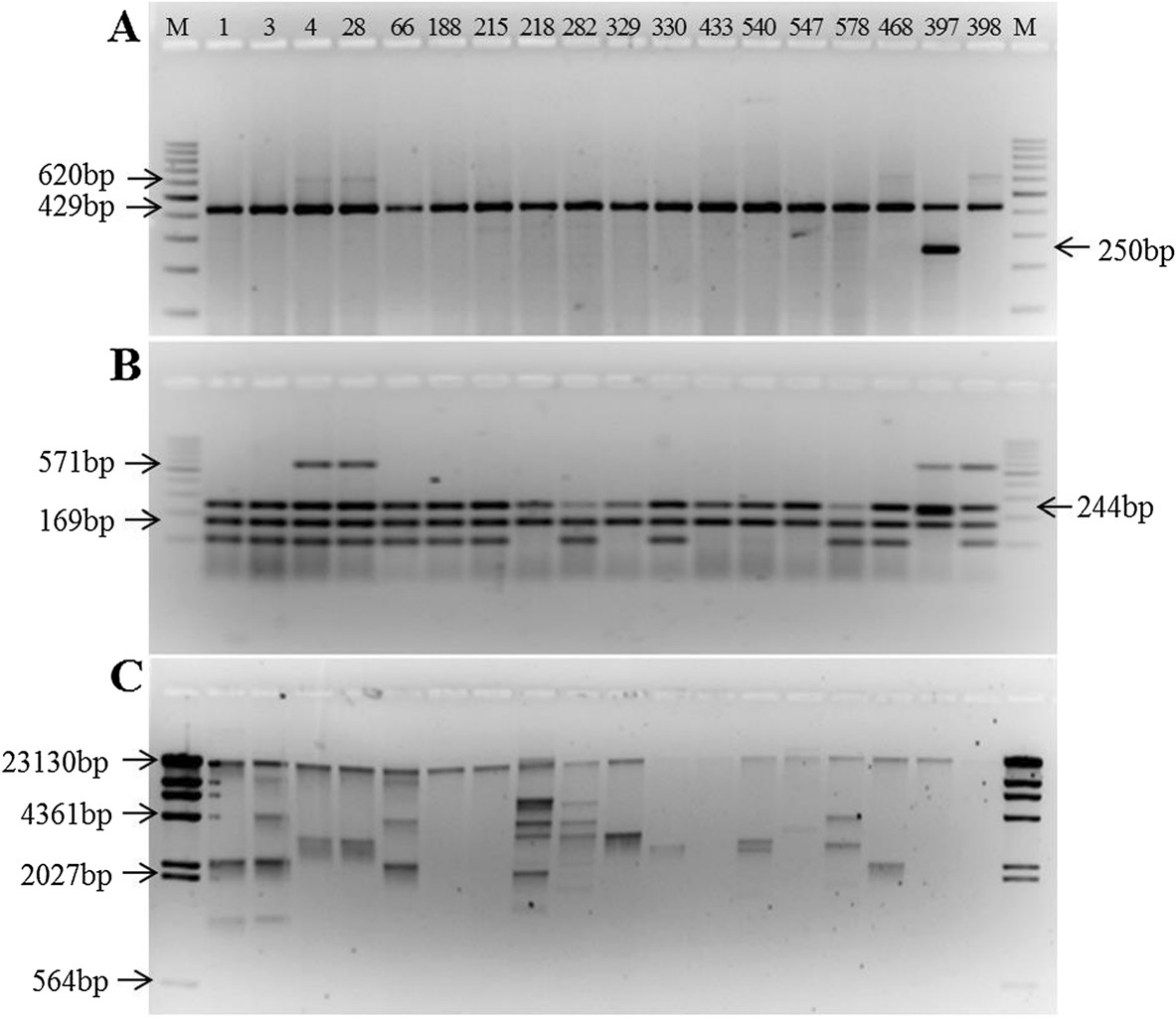
**Image of agarose gels for PCR identification of** ***Salmonella*** **spp. isolates from sewage sludge, presence of virulence genes and plasmidial profile. A**- Identification of *Salmonella* spp. and serotypes *Salmonella* Typhimurium e *Salmonella* Enteriditis; **B**- Detection of virulence genes; **C**- Plasmidial profile; Strains 397 and 398: positive control for *Salmonella* Enteriditis and *Salmonella* Typhimurium, respectively; **M**- molecular weight markers.

Figure 1 presents the image of agarose gels for PCR identification of *Salmonella* spp. previously isolated which were obtained from sewage sludge. In these strains, the presence of virulence genes and plasmidial profile were analysed. The identification of 40 isolates by PCR resulted in 36 strains containing the 429 bp band expected for all organisms identified as *Salmonella* spp. Three presented the 429 bp band in addition to the 620 bp band expected for *Salmonella* Typhimurium*. Salmonella* Enteritidis was not identified in this study, and four isolates were not identified as belonging to *Salmonella* group. As for plasmids, these extracromossomal elements were observed in 14 (40%) of the Salmonella strains subjected to serotyping, including *Salmonella* Typhimurium.

## Discussion

This study aims to present the level of *Salmonella* contamination in sewage sludge from WWTPs located in the most populated Brazilian metropolitan area. Data concerning the presence and concentration of such pathogen in sewage sludge are scarce in Brazil. Thus, the results herein reported could constitute an informational dataset, which would be used in order to give further support to interventional measures developed by sanitary authorities and also to the process of establishing guidelines for sludge usage on agricultural fields.

None of the less, we have also observed a huge difference in *Salmonella* spp. concentration amongst the five WWTPs assessed. In WWTP 1, WWTP 3, and WWTP 5, lime stabilizations were performed, which, according to Bean *et al*. [21], increases pH values up to 13, thus possibly eliminating all bacteria. In WWTP 2 and 4 where lime stabilization was not performed, *Salmonella* spp. concentration was higher if in comparison with WWTP 1, 3, and 5.

It is quite common to find such differences among results concerning the presence or concentration of *Salmonella* spp. in sewage sludge worldwide, even in those which undergo the same treatment process (mesophilic anaerobic digestion). In the present study, the frequency of positive samples were above DL (<0.006473 MNP/g) in 38.9% (21/54) of the samples analyzed, which was below the figures derived from other studies [22] and [23], which registered 92 - 100% (n =91) and 58% (n =24), respectively. The highest concentration of *Salmonella* spp. was found in WWTP 2 (1.2 × 10^1^ MPN/g), which is in agreement with the review previously carried out by Sidhu and Toze [3], wherein the concentration usually varied from 1.1 × 10^1^ to 5.9 × 10^3^ MPN/g TS. Wong *et al*. [24], on the contrary, found lower concentrations than the ones from the above mentioned studies, that is, ranging from 0.487 MPN/4 g to 0.954 MPN/4 g (0.122 – 0.238 MPN/g) in sludge from a WWTP in Michigan (USA). Such differences may be explained by the complex chemical sludge composition which differs from one country to another, type of treatment applied, regrowth or the presence of growth inhibitory factors, and population epidemiological profile [23]. Although distinct epidemiological studies have indicated the presence of *Salmonella* Enteritidis in sewage sludge, in the present study such serotype has not been detected. The absence of such specific serotype could be explained due to the limited number of strains serotyped. Similar results were noticed in Sweden [23]. Salström, in 2006, stated that such results could be due to the fact that this serotype is best adapted to human and animal hosts rather than to stressful environmental conditions [23]. None of the less, the presence of several serotypes in sewage sludge after outbreaks related to the ingestion of raw fruits and vegetables, such as *S.* Infantis, *S.* Newport, *S.* Javiana, *S.* Oranienburg, *S.* Mbandaka, *S.* Anatum, *S.* Müenchen, and *S.* Typhimurium [9,25-27], was observed. Furthermore, serotypes *S.* Newport and *S.* Typhimurium have a remarkable capacity to form a biofilm on leaves [28] and then to invade various tissues of plants [29].

The presence of *spvC* gene was found in only two out of the three *Salmonella* Typhimurium isolated strains, harbouring also *invA* and *sseL* genes. Peterson *et al*. [30] reported that up to that moment, the *spvC* gene had been found only in five *Salmonella* strains: *S.* Cholerasuis, S. Dublin, *S.* Enteritidis, and *S.* Typhimurium. Thus, the *Salmonella* Typhimurium strains mentioned in this study could represent a threat, as three virulence genes were detected even after the sewage sludge treatment.

From the 35 strains tested, 33 harboured *sseL* as well as the *invA* virulence genes, however two strains (*Salmonella* Molade and *Salmonella* München), did not harbour the latter virulence gene. The *invA* gene is widely recognised as a genetic marker for *Salmonella enterica* strains, as so can be found in almost all strains, being even used for PCR identification in this group [31]. Instead, upon assessing 630 different *Salmonella enterica* strains, Rahn *et al*. [32], found that 99.4% of those strains harboured the *invA* gene. Therefore, although found in the group related to the present study, the presence of *invA* gene is not universal.

Forty per cent of the 35 strains identified in this study presented plasmids (Table 2). According to Rychlik *et al*.[33], high molecular weight plasmids (above 20 Kb) are usually related to virulence genes or as resistance to antimicrobial agents; and low weight plasmids have not yet been fully comprehended, however, some of them could be related as resistant to heavy metals and also to other substances found in the environment. Although the isolation techniques performed in our study do not distinguish between high and low molecular weight plasmids, it was possible to observe the presence of such elements in 40% of the strains, including two *Salmonella* Typhimurium, which presented the *spvC* gene. The existence of plasmids in *Salmonella* strains in this study might come as a result in regard to the presence of heavy metals and high sulphur concentrations that are commonly present in São Paulo wastewaters and sewage sludge [34,35].

Concerning antimicrobial resistance, out of the five strains which were resistant to two or more drugs, four harboured plasmids, and one *Salmonella* Agona strain was resistant to two antimicrobial agents, presenting no plasmid at all (Table 2). Singh *et al*. [36] perceived that the presence of plasmids is not mandatory for the occurrence of antimicrobial drug resistance in *Salmonella* Enterica strains, once such resistant genes can also be found in chromosomes.

The results demonstrated in this research show that 14.2% (3/35) of the strains were resistant to two antimicrobial agents, and 5.7% (2/35) were MDR. Similar results were found by Sahlström *et al*. [23] – where 12% (12/101) of *Salmonella* isolates were resistant to at least one antimicrobial agent, and 7% (7/101) were MDR strains. Such results are quite different from those related to other studies previously conducted in Brazil, where *Salmonella* strains from human and non-human sources presented higher resistance to antimicrobial agents. In Rio de Janeiro, Fonseca *et al*. [37] isolated 35 *Salmonella* Infantis samples derived from stools of hospitalized children and found that 34 strains were resistant to at least two antimicrobial agents.

Studies conducted by Oliveira *et al*. [38] and Medeiros *et al*. [20] found in a farm strains of *Salmonella* in animal waste which were resistant to antibiotics, hence demonstrating that such kind of strain is circulating freely in the environment, and such fact can be considered as a crucial concern in case sewage sludge is used in agriculture.

Although the present study demonstrates a vast number of susceptible strains, the presence of an ESBL-producing and ciprofloxacin-resistant *Salmonella* (Agona) is worrisome. Since resistance to this antibiotic has increased and so has the production of ESBL [39], and also seeing that such is the alternative antibiotic with the aim of controlling MDR strains, treatment options are now limited.

Eventually, we also ought to take into account that some studies have presented the ability *Salmonella* spp. presents when it comes to regrowth in soil, internalization phenomena and its growth within plants tissues, as well as its capacity to survive for long periods in soil and crops [40-42]. Therefore, its presence must be considered as a major concern, even if such is found in low concentrations.

## Conclusions

The results obtained in this study demonstrate that several genres of *Salmonella* are circulating in the environment, including MDR strains, therefore they can easily be found in sewage sludge. Although *Salmonella* Enteritidis strains were not isolated in this study, the serotypes herein mentioned are implicated in several worldwide outbreaks related to these bacteria. Moreover, certain virulence factors were found in three samples of *Salmonella* Typhimurium which harboured *invA*, *sseL* and *spvC*. Therefore in order to avoid the spreading of such bacteria through the consumption of fresh products the application of sewage sludge in agricultural soils should only be allowed with the implementation of sanitary barriers, under the supervision of environmental and health authorities, such as different types of sewage sludge stabilizations, already present in other countries.

## Methods

### Sampling

Sludge samples were collected from five Wastewater Treatment Plants (WWTP) located in a Brazilian densely populated metropolitan region. The treatment process which was used in all WWTPs was activated sludge with mesophilic anaerobic sludge digestion. Table 3 shows the operational characteristics of each WWTP provided by Sabesp [43], such as sewage flow rates (L/s), generated sludge (Ton/d), and chemical conditioning.

**Table 3:** **Sewage flow rates (L/s), generated sludge (Ton/d), and chemical conditioning applied to the five WWTPs**

**WWTP**	**Sewage flow (L/s)**	**Generated sludge (Ton/d)**	**Chemical conditioning**
1	1.89	69.0	Lime + Ferric chloride
2	10.19	316.0	Ferric chloride + polymers
3	2.48	73.7	Lime + Ferric chloride + Polymers
4	0.80	14.3	Ferric chloride + polymers
5	0.85	41.0	Lime + Ferric chloride

Sampling was performed monthly in each WWTP in accordance with EPA’s recommendations [44] from January 2011 to December 2011. Samples (n = 54) were collected from a sludge pile after the dewatering process and stored in sterile plastic bottles which were kept chilled during transportation and analyzed within a 24-hour period.

### *Salmonella* spp. isolation

*Salmonella* spp. isolation and enumeration were performed according to the EPA Method 1682 [44]: *Salmonella* in Sewage Sludge (Biosolids) Modified Semisolid Rappaport-Vassiliadis (MSRV). This method is based on “the most probable number technique” (MPN). Briefly, prior to inoculation, 30 g of sewage sludge was diluted with buffered water and homogenized in a mixer for two minutes. Subsequently, volumes of 20, 10, and 1 mL were inoculated in three series of five tubes containing Tryptic Soy Broth Medium (TSB) (Difco®, Detroit, MI,USA) and incubated at 36°C for 24 hours.

Subsequent to incubation, six drops (30 μL) from each tube were transferred to selective MSRV medium (Difco®, Detroit, MI, USA) added with novobiocin (Difco®, Sparks, MD, USA) and malachite green in order to inhibit non-*Salmonella* species while permitting most *Salmonella* species to grow. MSRV plates were incubated at 42°C for 18 hours. Thereafter, colonies with “whitish halo” were transferred to Xylose-Lysine Desoxycholate agar (XLD) (Difco®, Detroit, MI, USA) and incubated at 36°C for 24 hours. The existence of typical colonies could be confirmed by the usage of Lysine-Iron agar (LIA) (Difco®, Detroit, MI, USA), Triple Sugar iron agar (TSI) (Difco®, Detroit, MI, USA), and of urea broth, followed by positive serological typing through the usage of poly O and poly H antisera. Determination of total solids (% dry weight) was performed according to USEPA [44] and used to calculate MPN/g dry weight. *Salmonella* spp. density was reported in MPN/4 g of dry weight.

In order to determine circulating serotypes, 40 *Salmonella* spp isolated strains representing all samples previously examined were selected from both lowest and highest dilution of all positive samples from the five WWTPs. For serotyping, the strains were sent to the Enteropathogens Laboratory from “Instituto Adolfo Lutz”, which is a branch of São Paulo State Department of Health.

### *Salmonella* spp. PCR identification

From the 307 isolates with positive serological reaction, 40 were further tested through PCR. DNA was extracted according to the method described by Chapman *et al*. [45] and amplification was performed with the set of primers described by Soumet *et al*. [46].

Briefly, amplifications were performed on 5 μl of the DNA samples which were previously extracted. Those samples were added to a mix (20 μl) consisting of 5 μl of 5× Gotaq buffer, 0.6 μmol of each primer (Invitrogen Corp – California USA), 200 μmol of dNTP (Thermo Fisher Scientific Inc.), and 1U of GO*Taq* Polymerase (Promega Inc.- Madinson, WI). Three sets of primers were used: ST11-ST15, specific for genus *Salmonella*; S1-S4 specific for *Salmonella* Enteritidis virulence genes; and Fli15-Typ04, which is a specific primer used to identify *fliC* genes only found in *Salmonella* Typhimurium. PCR reactions were carried out in a Mastercycler Thermocycler (Eppendorf - Hamburg, Germany). Primer sequences are described in Table 4.

**Table 4:** **PCR Primers were used for** ***Salmonella*** **spp. identification and virulence genes detection**

**Target**	**Primers**	**Primers sequence**	**Band size**	**Reference**
**Identification**	ST11	GCCAACCATTGCTAAATTGGCGCA	429 pb	[38]
	ST15	GGTAGAAATTCCCAGCGGGTACTGG		
	Fli15	CGGTGTTGCCCAGGTTGGTAAT	620 pb	
	Typ04	ACTCTTGCTGGCGGTGCGACTT		
	S1	GCCGTACACGAGCTTATAGA	250 pb	
	S4	ACCTACAGGGGCACAATAAC		
**Virulence**	SPVC-1	ACTCCTTGCACAACCAAATGCGGA	571 pb	[25]
	SPVC-2	TGTCTTCTGCATTTCGCCACCATCA		
	INVA-1	ACAGTGCTCGTTTACGACCTGAAT	244 pb	
	INVA-2	AGACGACTGGTACTGATCGATAAT		
	*sseL* F	TTCCGCGACAACCGACCTTTCTAA	169 pb	
	*sseL* R	TTCTTGAACCAGACCTTGCGTTGC		

ST11/ST15-amplifying sequences of *Salmonella* sp; S1/S4-amplifying sequences of a *Salmonella* Enteritidis phage type 4 strain; Fli15/Typ04-amplifying sequences of *Salmonella* Typhimurium strain.

### Detection of virulence genes

The 40 previously selected isolates were tested in order to find the presence of s*pvC*, *invA,* and s*seL* virulence genes, which are in accordance to Peterson *et al*. [30]. Within a 25 μl reaction volume, the following quantities were added: 5 μl of DNA template, 5 μl of 5× Gotaq buffer, 0.3 μmol of each primer (Bioneer Oligo Synthesis Report - Korea), 200 μmol of dNTP (Thermo Fisher Scientific Inc.), and 1U of GO*Taq* Polymerase (Promega Inc.- Wisconsin -USA) (Table 4).

### Electrophoresis

PCR products were performed by electrophoresis using a 3.0% agarose gel prepared with 1× TAE buffer and stained with ethidium bromide. A 100 bp DNA Ladder Plus (Thermo Fisher Scientific Inc.) was included as a size marker. Gel images were obtained via UV transillumination and captured by a gel documentation system (Epi Chemi II Darkroom and Software Labworks – UVP).

### Plasmid detection

Plasmid extraction was carried out by using a Wizard PlusSV Minipreps DNA Purification System commercial kit (Promega Inc.- Wisconsin -USA) using 2 mL of overnight culture according to the manufacturer’s instructions. Plasmids were recovered in 100 μL of nuclease-free water and stored at 20°C. Prior to plasmid separation by electrophoresis, the material was heated for 15 minutes at 65°C and then maintained in ice bath for five minutes. The same process was conducted with a Hind III marker.

Plasmids were undergone electrophoresis in 1.0% agarose gel for 3 hours at 3.0 V/cm along with a molecular Hind III weight marker (Thermo Fisher Scientific Inc.). The gel was stained by using ethidium bromide (10 mg/ml), visualised under ultraviolet light and captured by Epi Chemi II Darkroom, and UVP BioImaging Systems.

### Antimicrobial susceptibility testing

The 40 selected *Salmonella* spp. isolates were submitted to antimicrobial susceptibility tests by using the Kirby-Bauer disk-diffusion technique according to CLSI [47] recommendations. The antibiotics used were as follows: ciprofloxacin (5 μg); tetracycline (30 μg); trimethoprim-sulfamethoxazole (25 μg); cefotetan (30 μg); cefotaxime (30 μg); ceftazidime (30 μg); cefepime (30 μg); aztreonam (30 μg); imipenem (10 μg); meropenem (10 μg); and ertapenem (10 μg). In the case of resistance to β-lactams, further tests for enzyme production were performed [48]. Interpretation of inhibition zones was carried out by using CLSI [49] criteria.
